# The Antipsychotic Olanzapine Interacts with the Gut Microbiome to Cause Weight Gain in Mouse

**DOI:** 10.1371/journal.pone.0115225

**Published:** 2014-12-15

**Authors:** Andrew P. Morgan, James J. Crowley, Randal J. Nonneman, Corey R. Quackenbush, Cheryl N. Miller, Allison K. Ryan, Molly A. Bogue, Sur Herrera Paredes, Scott Yourstone, Ian M. Carroll, Thomas H. Kawula, Maureen A. Bower, R. Balfour Sartor, Patrick F. Sullivan

**Affiliations:** 1 Departments of Genetics, University of North Carolina at Chapel Hill, Chapel Hill, North Carolina, United States of America; 2 Department of Microbiology and Immunology, University of North Carolina at Chapel Hill, Chapel Hill, North Carolina, United States of America; 3 Department of Biology, University of North Carolina at Chapel Hill, Chapel Hill, North Carolina, United States of America; 4 Department of Medicine, University of North Carolina at Chapel Hill, Chapel Hill, North Carolina, United States of America; 5 Curriculum in Bioinformatics and Computational Biology, University of North Carolina at Chapel Hill, Chapel Hill, North Carolina, United States of America; 6 The Jackson Laboratory, Bar Harbor, Maine, United States of America; 7 Center for Gastrointestinal Biology and Disease, University of North Carolina at Chapel Hill, Chapel Hill, North Carolina, United States of America; 8 Department of Psychiatry, University of North Carolina at Chapel Hill, Chapel Hill, North Carolina, United States of America; Charité, Campus Benjamin Franklin, Germany

## Abstract

The second-generation antipsychotic olanzapine is effective in reducing psychotic symptoms but can cause extreme weight gain in human patients. We investigated the role of the gut microbiota in this adverse drug effect using a mouse model. First, we used germ-free C57BL/6J mice to demonstrate that gut bacteria are necessary and sufficient for weight gain caused by oral delivery of olanzapine. Second, we surveyed fecal microbiota before, during, and after treatment and found that olanzapine potentiated a shift towards an “obesogenic” bacterial profile. Finally, we demonstrated that olanzapine has antimicrobial activity *in vitro* against resident enteric bacterial strains. These results collectively provide strong evidence for a mechanism underlying olanzapine-induced weight gain in mouse and a hypothesis for clinical translation in human patients.

## Introduction

The second-generation (“atypical”) antipsychotic drug olanzapine is commonly prescribed as a first-line treatment for schizophrenia, bipolar disorder, and other psychotic disorders. Since their introduction in the 1990s, olanzapine and other atypical antipsychotics have come into widespread clinical use because they are efficacious and usually lack the extrapyramidal adverse drug reactions associated with older, “typical” antipsychotics like haloperidol [1], [2]. However, atypical antipsychotics can induce dramatic weight gain: 11–17 kg in a systematic review of treatment-naïve adults [3] and 7–9 kg in adolescents over short-term treatment [4]. Olanzapine in particular is associated with a deterioration of metabolic parameters and development of a syndrome resembling type 2 diabetes [3]–[6].

Individuals with schizophrenia are less physically healthy than the general population, with life expectancy reduced by as much as 25 years [3], [7]–[9]. Cardiovascular disease is a leading cause of death in schizophrenia [3], [9]: tobacco use, poor diet, and socioeconomic disparities conspire to create a profoundly unfavorable cardiovascular risk profile that is compounded by weight gain and the metabolic side effects of antipsychotic drugs. Understanding the mechanisms [10]–[13] of these adverse drug reactions and developing mitigating strategies are of considerable clinical importance [14].

The role of the commensal microbiome of the mammalian gut in health and disease has become a topic of intense study. Bacterial concentrations can be as high as 10^11^ cells/mL in the distal gut, encompassing a gene repertoire 150 times larger than that of the human host [15] with broad-ranging metabolic capacity [16], [17]. Millions of years of coevolution have shaped the metabolic capacity of the gut microbiome and its human host [18]. Recent studies in animal models and humans have demonstrated a causal role for the gut microbiome in obesity [19]–[24], which is mediated by complex interactions with host environment [25], [26], diet [21], [27] and genetics [25], [28].

Since olanzapine is usually administered orally and approximately 30% of the dose enters the enterohepatic circulation [29], the gut microbiome represents a plausible mechanistic link and potential therapeutic target for olanzapine-induced metabolic dysfunction. After establishing that C57BL/6J mice gain considerable weight while consuming olanzapine, we demonstrated that: (*a*) germ-free mice do not gain excess weight on olanzapine, (*b*) the same germ-free mice gain excess weight on olanzapine following introduction of cecal microbiota, (*c*) olanzapine induces a shift to an obesogenic gut bacterial profile, and (*d*) olanzapine has modest intrinsic antimicrobial activity. Our results are broadly consistent with those recently obtained in outbred rats [30], [31] but offer a more precise characterization of inter-individual variability against a defined genetic background.

## Materials and Methods

### Olanzapine administration

Olanzapine was compounded into high fat (45 kcal%) food (diet D09092903) at a concentration of 50 mg/kg of diet by Research Diets, Inc (New Brunswick, NJ). This dose was selected following a four-week dose-ranging study in C57BL/6J mice (0, 12.5, 25, 50, 100 mg/kg), since it produced steady-state plasma levels (21±5 ng/ml) closest to the clinically relevant range (10–50 ng/mL; see **S2 Figure** and [32]). Plasma olanzapine levels were measured using the liquid chromatography/tandem mass spectrometry (LC-MS/MS) method of Zhang *et al*. [33]. Placebo-treated animals received the same high fat diet (D09092903), minus olanzapine, prepared in parallel to control for batch effects.

### Strain survey

**Figure 1:**
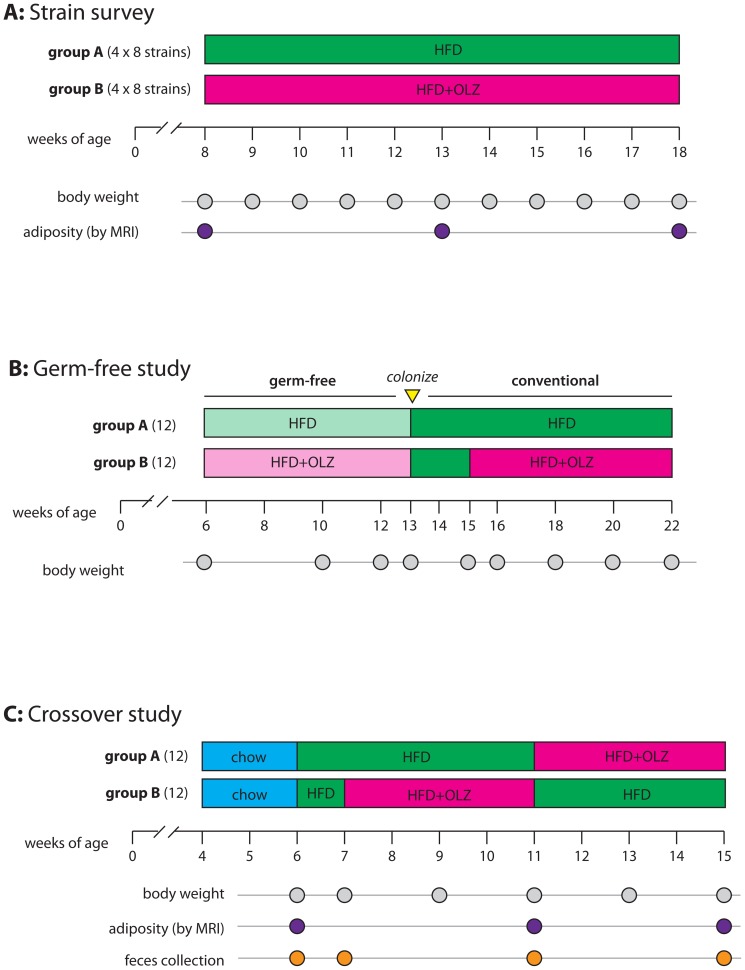
Experimental design. (**A**) Eight groups of 8 female mice representing the founder strains of the Collaborative Cross project (A/J, C57BL/6J, 129S1SvlmJ, NOD/ShiLtJ, NZO/HlLtJ, CAST/EiJ, PWK/PhJ, WSB/EiJ) were received from the Jackson Laboratory at 6 weeks of age. Within each strain, mice were randomized to receive either olanzapine (OLZ, 50 mg/kg diet) or placebo while consuming a high-fat diet (HFD) *ad libitum* beginning at 8 weeks of age. Body weight was measured at time points indicated by grey dots; adiposity was measured by magnetic resonance imaging (MRI) at time points indicated by purple dots. (**B**) Six cages of 4 female C57BL/6J mice were reared in germ-free (axenic) conditions. At 6 weeks of age, each cage was randomized to either HFD alone (3 cages; *n* = 12) or HFD plus OLZ (3 cages; *n* = 12). After 7 weeks of treatment mice were inoculated by oral gavage followed by a 2-week drug-free colonization period. Treatment was resumed for an additional 7 weeks under conventional housing conditions. (**C**) Two groups of 12 female C57BL/6J mice were received from the Jackson Laboratory at 4 weeks of age and randomized to study arms (group A or B). Both groups were acclimated to the facility for 2 weeks, during which time they were fed regular mouse chow, then both groups were switched to HFD for 1 week. For weeks 8–11, group A was maintained on high-fat diet and group B was switched to HFD plus OLZ. For weeks 12–15, groups were swapped so that group A received OLZ and group B received no drug. Body weight and adiposity phenotypes were collected at the indicated time points. Fecal pellets were collected for microbiome profiling at time points indicated by orange dots.

For the strain survey (**Fig. 1A**), *n* = 8 female mice (aged 6 weeks) from each of eight inbred strains (A/J, C57BL/6J, 129S1SvlmJ, NOD/ShiLtJ, NZO/HlLtJ, CAST/EiJ, PWK/PhJ, WSB/EiJ) were acquired from the Jackson Laboratory (Bar Harbor, ME). All mice were maintained on standard chow (Purina Prolab RMH3000, 14 kcal% fat) until 8 weeks of age and then switched to high fat diet ± olanzapine for 10 weeks. There was one cage of olanzapine-treated mice (*n* = 4) and one cage of placebo-treated mice (*n* = 4) for each strain. Animals were maintained on a 14-hour light, 10-hour dark schedule with lights on at 0600. The housing room was maintained at 20–24°C with 40–50% relative humidity. Mice were group-housed (4 per cage) in standard 20 cm×30 cm ventilated polysulfone cages with laboratory grade Bed-O-Cob bedding. Water and feed were available *ad libitum* throughout the experiment.

### Germ-free study

Germ-free experiments were performed in the National Gnotobiotic Rodent Resource Center at the University of North Carolina. Twenty-four female C57BL/6J mice were born in a germ-free isolator and randomized to 6 cages (4 per cage.) At 6 weeks of age, 3 cages were randomized to receive olanzapine and 3 cages to receive placebo (*n* = 12 mice per group; **Fig. 1B**). Olanzapine was administered in feed as detailed above, except that both drug and placebo feed was doubly irradiated prior to import into the germ-free isolator. Germ-free status was confirmed by biweekly monitoring for the presence of bacteria in the feces by aerobic and anaerobic culture as well as Gram staining of the stool. After 7 weeks of treatment, mice were moved to conventional housing (maintaining the same cage assignments) at which time each mouse was gavaged with 200 caecal slurry (500 uL caecal contents form a single conventionally-raised C57BL/6J female diluted 1∶10 in sterile PBS)[34]. Following gavage, all mice were maintained on drug-free high-fat diet for 2 weeks in order to allow colonization of the gut without interference from olanzapine. After the 2 week colonization period, treatment was resumed in the olanzapine group for another 7 weeks.

### Crossover study

For the crossover study (**Fig. 1C**), *n* = 24 female C57BL/6J mice (aged 4 weeks) were acquired from the Jackson Laboratory. Upon arrival in the facility, animals were randomly assigned to cages (4 mice per cage), and cages randomized to study arms (group A or group A; 3 cages per group). Both groups were fed standard chow from 4–6 weeks of age, then both groups were switched to high-fat diet for 1 week. For weeks 8–11, group A received placebo and group B received olanzapine. During weeks 12–15, groups were swapped so that group A received olanzapine and group B received no drug. All mice received the high-fat diet from week 7 to the conclusion of the study.

### Collection of metabolic phenotypes

Body weight (±0.1 g) was measured weekly for the strain survey, biweekly in the germ-free isolator, and biweekly for the crossover study, always between the hours of 0800–1000. Body composition (fat, lean, and water mass ±0.01 g) was measured in un-anaesthetized mice using a quantitative magnetic resonance body composition analyzer [35] (EchoMRI, Houston, TX, USA). Adiposity (% body fat) was calculated as 100× (fat weight)/(total body weight).

### Ethics statement

This study was carried out in strict accordance with the recommendations in the Guide for the Care and Use of Laboratory Animals of the National Institutes of Health. The protocols were approved by the Institutional Animal Care and Use Committee of the University of North Carolina at Chapel Hill (protocol numbers: 11–318.0, 11–320.0).

### Bacterial growth-inhibition assays

*Escherichia coli* NC101 and *Enterococcus fecalis* OGIRF were grown shaking overnight at 37°C in brain heart infusion (BHI) broth supplemented with 1% IsoVitalex (Becton-Dickson). Overnight cultures were resuspended in phosphate-buffered saline (PBS) to a Klett reading of 100 (approximately 1×10^9^ CFU/ml). Resuspended cultures were diluted 1∶20 in a 96 well plate with a range of olanzapine concentrations (0–580 *µ*g ml^−1^). Olanzapine is insoluble in BHI so DMSO was added and used as a control (17.5% of the culture volume). Growth curves were performed at 37°C while shaking in an Infinite M200 (Tecan) apparatus in 96-well microtiter plates with absorbance (optical density at 600 nm, OD_600_) monitored every 15 min. Minimal inhibitory concentrations were determined by identifying the lowest concentration of olanzapine that inhibited growth and did not exceed an OD_600_ of 0.2.

### Gut microbiome sampling

Fecal samples were collected from the 24 mice in the crossover at 4 different time points (**Fig. 1C**, a total of 96 samples), in order to follow individual changes in the microbiome resulting from diet and drug treatment. Fresh fecal pellets were collected by transferring an individual mouse to a clean cage for 5 minutes, collecting 3–5 pellets into a microcentrifuge tube with sterile forceps, snap freezing on dry ice and storing at −80°C. Bacterial DNA was extracted using a PowerSoil 96-well DNA isolation kit (MO BIO Laboratories, Inc; Carlsbad, CA) on an automated pipetting system (epMotion 5075; Eppendorf AG; Hamburg, Germany).

Hypervariable region 4 (V4) of the 16S ribosomal RNA subunit gene was amplified by the molecule-tagged PCR method [36] Briefly, this technique adds a unique oligonucleotide sequence (the “molecular tag”) to each template molecule in the first round of amplification so that PCR artifacts are readily identified and corrected in downstream analyses. A 96-plex barcoded library was prepared using the Illumina Nextera chemistry (Illumina Inc; San Diego, CA). Samples were randomly allocated to barcodes. The library was sequenced in a single 2×250 bp paired-end run on the Illumina MiSeq platform. Standard preprocessing and demultiplexing of sequence reads was performed using the CASAVA v1.8.2 software from Illumina. Library preparation, sequencing and preprocessing were performed by staff at the University of North Carolina High-Throughput Sequencing Facility. Raw sequence reads have been deposited in the NCBI Short Read Archiveunder BioProject PRJNA264871.

Sequence reads were processed using the MT_MTToobox software of Lundberg *et al*. (https://sites.google.com/site/moleculetagtoolbox). This pipeline merges raw reads into consensus sequences representing a single template (via its molecular tag), thus detecting and removing PCR chimeras and amplification artifacts; counts read abundance within each consensus group; and bins consensus sequences into operational taxonomic units (OTUs) at a 97% sequence similarity threshold. OTUs were assigned a taxonomic identity with the RDP classifier [37] trained on the most recent release (4 February 2011) of the Los Alamos National Laboratory's GreenGenes taxonomic reference (gg_97_otus_4feb2011), using the scripts in the QIIME v1.5.0 package [38]. Consensus sequences for the OTUs were aligned using PyNAST [39] (via QIIME) guided by the aforementioned GreenGenes reference alignment, and a corresponding phylogeny inferred using FastTree [40] (via QIIME). Between-samples (*β*) diversity indices were calculated using UniFrac [41] (via QIIME) after rarefying all samples to 6997 unique template sequences. The final UniFrac distance matrices represent the average over 10 000 rarefactions.

### Statistical analysis

In order to account for the several levels of correlation present in this experiment, we analyzed changes in body weight, adiposity and microbial parameters using generalized linear mixed models (GLMMs) as implemented in the R package lme4 (http://lme4.r-forge.r-project.org). Between-group differences in overall microbiota composition were assessed by PERMANOVA [42] as implemented in the R package vegan (http://vegan.r-forge.r-project.org), and visualized using non-metric multidimensional scaling as implemented in the R package MASS [43]. Further details regarding statistical methods are provided in **S1 Notes**.

## Results

### Olanzapine potentiates weight gain on high-fat diet

To develop a model for olanzapine-induced weight gain in mouse, we surveyed eight inbred strains (A/J, C57BL/6J, 129S1SvlmJ, NOD/ShiLtJ, NZO/HlLtJ, CAST/EiJ, PWK/PhJ, WSB/EiJ) which are the founder strains of the Collaborative Cross population [44]. Eight females from each strain were randomized to receive either olanzapine (compounded into feed at 50 mg/kg) or placebo and were fed a high-fat diet *ad libitum* (**Fig. 1A**). The rate of weight gain and the effect of olanzapine were highly variable between strains (**S1A Figure**). Plasma olanzapine levels were measured after 18 weeks of treatment (**S1B Figure**). C57BL/6J mice were highly susceptible to olanzapine-induced weight gain, gaining a mean of 17.1% over initial body weight (95% CI 13.6–20.6%, *p* = 1.06×10^−5^) and were therefore selected for further study. A dose-response study was conducted in C57BL/6J mice order to titrate drug dosage to a clinically-relevant level (**S2 Figure**). The plasma concentration in which olanzapine has the greatest effect on body weight and adiposity (10–25 ng/mL) corresponds with the therapeutic range in human patients [32].

### Gut microbiota is necessary and sufficient for olanzapine-induced weight gain

**Figure 2:**
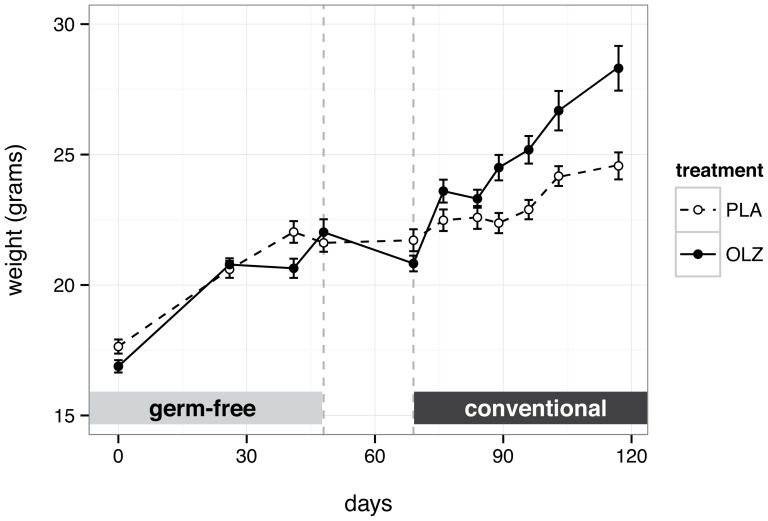
Effect of high-fat diet plus olanzapine (OLZ; filled circles) versus placebo (PLA; open circles) on body weight in germ-free mice. Points in the left-hand third represent observations under germ-free conditions. The first dashed line marks the beginning of the drug-free conventionalization period (with colonization by gavage) and the second dashed line marks the resumption of treatment under conventional conditions. Each point represents the mean across 12 mice; error bars are ± 2 standard error.

To investigate the role of the gut microbiota in olanzapine-induced weight gain, 24 female C57BL/6J mice were raised in germ-free conditions (**Fig. 1B**). At 6 weeks of age, 12 mice were randomized to high-fat diet plus olanzapine (50 mg/kg) and 12 to high fat diet alone. After 7 weeks of treatment in germ-free conditions, no significant difference in body weight was observed between groups (**Fig. 2**; 95% CI for difference −0.75–1.61 grams; *p* = 0.478). Under conventional conditions in the strain survey (**S1A Figure**), olanzapine-treated C57BL/6J females had gained 4.6 grams more than untreated mice (95% CI for difference 1.67–7.48 grams; *p* = 8.81×10^−3^) during the first 7 weeks of treatment. Mice were then transferred to conventional housing and gavaged with a slurry of cecal contents from a female C57BL/6J donor mouse. Following a 2-week acclimation period, treatment was resumed for another 7 weeks, during which the olanzapine group gained significantly more weight than placebo group (**Fig. 2**; 95% CI for difference 1.33–5.75 grams; *p* = 4.9×10^−3^). These results demonstrate that gut microbiota are both necessary and sufficient for weight gain induced by olanzapine in the context of a high-fat diet.

### Interrogating the gut microbiota and weight using a crossover design

To study the effects of olanzapine on the gut microbiome, 24 six-week-old female C57BL/6J mice raised on regular mouse chow in conventional conditions consumed a high-fat diet for 2 weeks, and then randomized to receive either 4 weeks of high-fat diet plus placebo (group A) or 4 weeks of high-fat diet plus olanzapine (group B). After 4 weeks the treatments were switched. Total body weight and adiposity were measured periodically throughout (**Fig. 1C**). Weight gain during the olanzapine phase (shaded interval in **S3A Figure**) was more rapid than during the placebo phase (95% CI 2.9–10.9% increase over initial weight, *p* = 2.80×10^−7^). The effects of olanzapine were variable between mice (**S4 Figure**). Adiposity (fat mass as a percentage of total body weight, assessed by whole body MRI) also increased markedly during the olanzapine phase (**S3B Figure**) and was highly correlated with total body weight (**S3C Figure**; Pearson's r = 0.894, 95% CI 0.836–0.933). Olanzapine was associated with increased adiposity even after accounting for weight gain (95% CI 1.4–4.5%, *p* = 4.46×10^−4^).

Fecal pellets were collected from each mouse before, during and after initiation of the high-fat diet and olanzapine treatment (4 temporally-matched samples per mouse, **Fig. 1C**). Gut microbiota were surveyed by high-throughput sequencing of the bacterial 16S ribosomal RNA gene, a widely-used marker gene in microbial ecology. The resulting sequences were clustered into a total of 8067 operational taxonomic units (OTUs) and were identified and classified at the family level, of which a median of 612 were present per individual sample. Further details of the sequencing protocol, analysis, and bioinformatics are provided in supplementary material.

### Olanzapine and high-fat diet act synergistically on gut microbiota

**Figure 3:**
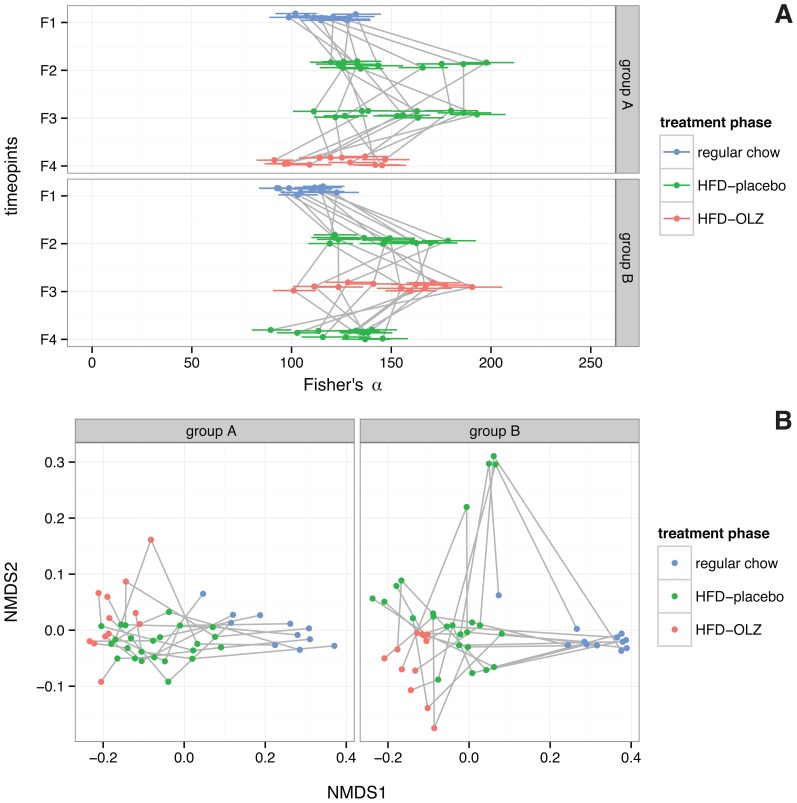
Effect of high-fat diet (HFD) and olanzapine (OLZ) treatment on diversity of gut microbiota during crossover study. (**A**) Within-subject (*α*) diversity, quantified by Fisher's *α* [45]; larger values indicate greater diversity (*n* = 95 samples  = 4 time points ×24 subjects – 1 failed sample). Panels are split by study arm (see **Error! Reference source not found**.) and points are colored according to diet and treatment status: on regular chow prior to initiation of HFD (regular), during treatment with placebo (HFD-placebo) or during treatment with OLZ (HFD-OLZ). Each point represents a single individual and bars indicate 95% confidence intervals about the point estimates; observations on the same subject are connected by a grey line. (**B**) Between-subjects (*β*) diversity (*n* = 95 samples  = 4 time points ×24 subjects – 1 failed sample). Pairwise distances between samples were calculated using weighted UniFrac and visualized using non-metric multidimensional scaling along two axes, which together explain 98% of between-subjects variance. Observations on the same subject are connected by a grey line.

The switch to high-fat diet was associated with a modest increase in within-sample microbial diversity (*α*-diversity) after adjusting for temporal and co-housing effects (Fisher's *α* [45], 95% CI 23.5–43.1 additional units, *p* = 4.02×10^−8^), but this effect varied between mice (**Fig. 3A**). Olanzapine treatment decreased *α*-diversity, but the effect was not significant after adjusting for temporal and co-housing effects (*p* = 0.449).

Between-samples (*β*) diversity was measured by weighted and unweighted UniFrac metrics, complementary phylogenetically-scaled multivariate distance metrics [41]. In the weighted UniFrac analysis (**Fig. 3B**, which is sensitive to the presence/absence of OTUs and differences in abundance between samples, the high-fat diet effect accounts for 46.9% of variance (permutation *p*<1.00×10^−4^; **Table 1**), while the olanzapine treatment effect explains 2.4% (permutation *p* = 0.0028). Within-groups dispersion was not different in the regular chow *vs*. high-fat diet or high-fat diet-placebo *vs*. high-fat diet-olanzapine comparisons. Unweighted UniFrac results are presented in **S5 Figure** and discussed in supplementary material.

**Table 1:** PERMANOVA decomposition of β-diversity as measured by UniFrac.

	Term	df	SS^∧^	F^#^	*R* ^2^	*p*-value
*Weighted UniFrac*	cage	5	0.3537	3.843	0.0728	0.0004
	diet	1	2.2813	123.94	0.46954	<1.00×10^−4^
	time point	2	0.5435	14.762	0.11185	<1.00×10^−4^
	drug	1	0.1156	6.278	0.02378	0.0028
	Residuals	85	1.5646	0.32202		
	Total	94	4.8586			
*Unweighted UniFrac*	cage	5	1.279	1.944	0.07742	<1.00×10^−4^
	diet	1	2.7293	20.7412	0.16519	<1.00×10^−4^
	time point	2	1.096	4.1644	0.06634	<1.00×10^−4^
	drug	1	0.2325	1.767	0.01407	0.021
	Residuals	85	11.1849	0.67698		
	Total	94	16.5217			

*df, degrees of freedom; ^∧^ **SS**, Sum of squares; ^#^ **F**, pseudo-*F*-statistic.

**Figure 4:**
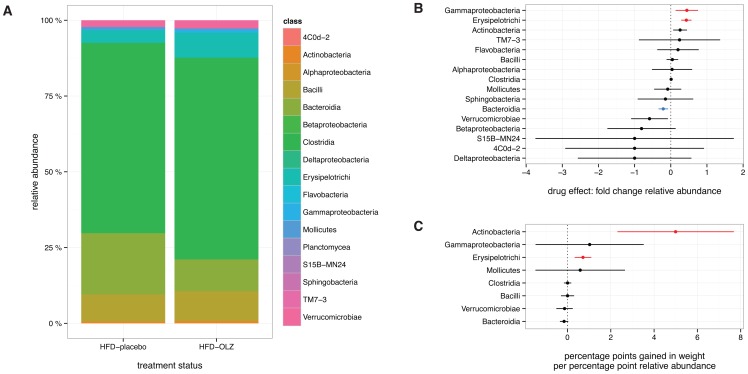
Compositional shifts in gut microbiota induced by high-fat diet (HFD) and olanzapine (OLZ) during crossover study. (**A**) Colored blocks show relative abundance of specific bacterial classes during treatment with OLZ (HFD-OLZ) or placebo (HFD-placebo), pooled across individuals in each of the two study arms (*n* = 3 time points ×24 subjects). (**B**) Effect of OLZ on relative abundance of specific taxa, expressed as confidence intervals (at nominal significance level *α* = 0.05) about the mean fold-change in relative abundance. Taxa with a statistically-significant increase are shown in red, and those with a significant decrease in blue. (**C**) Effect of relative abundance of specific taxa on weight gain, expressed as confidence intervals (at nominal significance level *α* = 0.05) about the effect estimate.

Gut microbiota composition for mice treated with olanzapine or placebo while receiving a high-fat diet is presented in **Fig. 4A** (in aggregate) and **S6** **Figure** (per individual). Olanzapine treatment induced an increase in the relative abundance of members of class Erysipelotrichi from phylum Firmicutes (95% CI 1.7–3.4%, adjusted *p* = 2.63×10^−6^) and class Gammaproteobacteria from phylum Proteobacteria (95% CI 0.079–0.45%, adjusted *p* = 3.11×10^−2^), and a decrease in class Bacteroidia from phylum Bacteroidetes (95% CI −5.3–−1.2%, adjusted *p* = 2.06×10^−2^) (**Fig. 4B**). This pattern has previously been associated with obesity in mouse and human [19], [24].

### Gut microbiota composition is correlated with weight gain

Relationship between relative abundance of specific bacterial taxa and weight gain during olanzapine treatment was assessed using linear mixed-effects models (**Fig. 4C**). The relative abundance of members of class Erysipelotrichi increased with olanzapine treatment and was associated with more rapid weight gain (0.71% increase in weight per 1% increase in abundance, 95% CI 0.33–1.10, *p* = 5.45×10^−3^). Class Actinobacteria, for which a drug effect was not noted, was also associated with weight gain (4.9% increase in weight per 1% increase in abundance, 95% CI 2.3–7.7, *p* = 5.44×10^−3^).

### Olanzapine has direct and specific antimicrobial action *in vitro*

**Figure 5:**
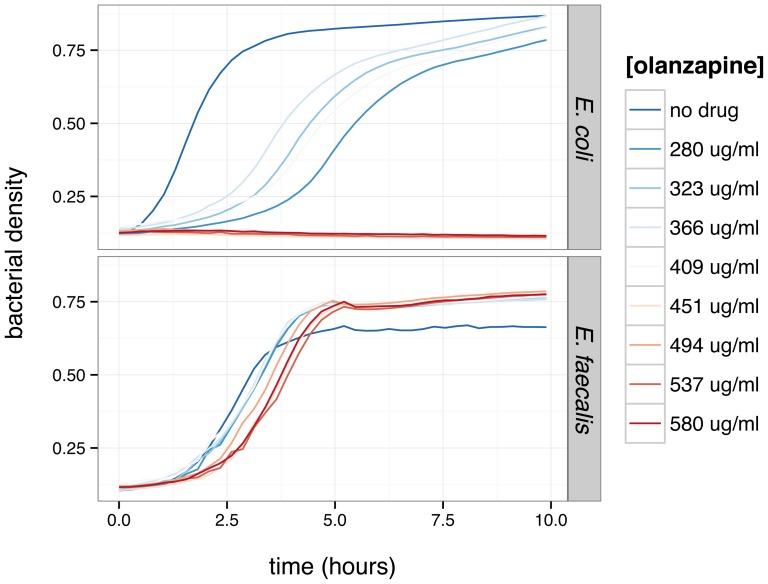
Effect of olanzapine (OLZ) on growth of *Escherichia coli* NC101 and *Enteroccoccus faecalis* OGIRF *in vitro*. Bacterial growth is measured as optical density at 600 nm wavelength. Measurements of growth in rich medium (brain heart infusion, BHI) plus vehicle (dimethylsulfoxide) only is included as a control. Each line represents the mean of three independent experiments.

We tested the effects of olanzapine on growth of two commensal enteric bacterial strains (*Escherichia coli* NC101 and *Enterococcus faecalis* OGIRF) across a range of supraphysiologic concentrations (280 *µ*g/mL to 560 *µ*g/mL) *in vitro*. These two strains were chosen because they represent highly abundant species from two of the dominant phyla (*E. coli*: Proteobacteria; *E. faecalis*: Firmicutes) of the mammalian gut. Susceptibility to olanzapine differs between the two species as shown in **Fig. 5**. Growth of *E. coli* is completely inhibited by 537 *µ*g/ml olanzapine, while growth of *E. faecalis* shows a delay in reaching log-phase growth but not complete growth arrest.

## Discussion

We have demonstrated that the antipsychotic olanzapine, when administered at a clinically-relevant dose, accelerates weight gain resulting from high-fat diet in C57BL/6J mice. This effect is absent under germ-free conditions but emerges quickly upon microbial colonization of the gut. Gut microorganisms are therefore necessary and sufficient for a common adverse effect of this highly-prescribed antipsychotic. Using a randomized crossover study design that accounts for inter-individual variation in the gut microbiota, we have shown that olanzapine induces subtle changes in the composition of the gut microbiota beyond the effect of a high-fat diet alone. Importantly, these alterations are qualitatively similar to the effect of a high-fat diet alone, and are quantitatively correlated with degree of weight gain. Finally and surprisingly, we find that olanzapine has antibiotic activity for two common resident bacterial species *in vitro*. To our knowledge, these results provide the first description of an adverse drug effect mediated by the commensal microbiome.

Weight gain was initially measured in the eight inbred mouse strains that are the founders of the Collaborative Cross [44], [46]. Consistent with observations in human studies, the survey revealed variation in both magnitude and reproducibility of olanzapine-induced weight gain between strains during high-fat feeding, independent of steady-state drug concentrations. This indicates that susceptibility to weight gain has a genetic component amenable to dissection via the Collaborative Cross or its sister resource, the Diversity Outbred stock [47].

The composition of the gut microbiota has been shown to be heritable and genetically complex [28]. In contrast to previous experiments in outbred rats [11], [30], [31], we studied isogenic mice. The degree of inter-individual variability observed in changes to the gut microbiota is therefore striking (**Fig. 3** **, S6 Figure**). A portion of this variability is probably technical artifact, [48] but true stochastic effects almost certainly make important additional contributions [49], which we detect as temporal and cage effects (**Table 1**). The large bloom in Verrucomicrobiae in three cages at the fourth fecal-sampling time point (**S6 Figure**) may be an example and is consistent with observations that the microbiota of co-housed mice tend to converge over time [25], [49]. Finally, the maternal microbiota and the post-weaning environment can influence the composition of the gut microbiota during the initial period of colonization [50]. Although we do not directly control for maternal effects, our use of careful randomization of mice to cages and to study arms minimized confounding due to early-life exposures.

The specific alterations in gut community composition that we attribute to olanzapine are similar to those attributed to high-fat diet [19], [21], [22], [30], [31]. This is immediately evident from the NMDS plot in **Fig. 3**: observations during placebo and olanzapine treatment phases vary along the same dimension as observations during feeding with regular chow and observations during high-fat feeding, although the effect of diet appears to predominate. That the effect sizes for both high-fat diet (46.9% *vs.* 16.5% variance explained) and olanzapine (2.4% *vs.* 1.4%) are greater in the weighted than unweighted UniFrac analyses (**Table 1**) suggests that drug- and diet-induced alterations in community structure are more quantitative than qualitative. Relative abundance of class Erysipelotrichi (phylum Firmicutes) and class Gammaproteobacteria (phylum Proteobacteria) increased during olanzapine treatment, while relative abundance of members of class Bacteroidia (phylum Bacteroidetes) decreased. An identical pattern has previously been associated with the switch from low-fat chow to a high-fat “Western diet” in mice with humanized gut microbiota [22]. Both Erysipelotrichi and Gammaproteobacteria have been associated with non-alcoholic fatty liver disease independently of weight gain in human [51], [52] and mouse [53]. Class Bacteroidia, decreased during olanzapine treatment in the present experiment and negatively (though not significantly) correlated with weight gain, has been shown to be enriched in lean members of twin pairs discordant for obesity, and its leanness-promoting effects were transmissible [24]. Collectively these associations indicate that olanzapine induces a shift towards a microbiota that has been demonstrated in both mouse and human to promote weight gain. However, we note that the composition of gut bacterial communities is an imperfect proxy for their metabolic capacity [50]; further studies will be required to elucidate functional consequences of olanzapine treatment.

Finally we demonstrate that olanzapine has direct antibacterial activity *in vitro* against two bacterial isolates (*E. coli* NC101 and *E. faecalis* OGIRF) derived from the mammalian gut (**Fig. 5**). Olanzapine undergoes enterohepatic recycling, and an autopsy report found that olanzapine was concentrated by one to two orders of magnitude in bile relative to the blood [54]. Local concentrations along the gut lumen – from the duodenum to the distal colon – may thus far exceed steady-state plasma levels. Although the microbial ecology of the gut is far too complex to allow robust predictions from experiments with two bacterial strains tested in isolation, our data supply proof of principle for the idea that olanzapine may directly perturb the luminal enteric community. The EC50 metric is suited to attempts to eradicate microbial pathogens; we hypothesize that therapeutic doses of olanzapine, while well below estimated EC50, still provide a subtle selective pressure that nudges the ecology of the gut towards an obesogenic composition. Indeed, other antipsychotic drugs and their metabolites have antimicrobial activity *in vitro*: for instance, chlorpromazine against *Mycobacterium tuberculosis* [55] and thioridazine against a methicillin-resistant *Staphylococcus aureus* isolate [56].

Germ-free animals provide a means for establishing causality in microbiome studies. The absence of excess weight gain with olanzapine administration under germ-free conditions demonstrates that the gut microbiota are required for this drug effect. The rapid appearance of a weight gap in the olanzapine-treated group following inoculation of all mice with cecal contents from a single donor demonstrates that the gut microbiota are sufficient for the drug effect. Complex bidirectional interactions occur between the brain, endocrine system, enteric nervous system, gut epithelium and microbiota in both rodents and humans [57], and olanzapine may perturb this “brain-gut-enteric microbiota” axis. Nonetheless, whether the causal pathway is direct or indirect, the enteric microbiota must mediate the effects of olanzapine on body weight.

In light of evidence that olanzapine has intrinsic antimicrobial activity, we propose that its actions in the gut may be analogous to the chronic low-dose antibiotic regimens used to promote weight gain in livestock. Such treatments have been shown to shape the composition and the metabolic capacity of the gut microbiota in mice, resulting in long-term changes in energy balance [23]. We suggest that olanzapine may promote weight gain and adiposity by a similar mechanism, and that inter-individual variability in composition of the gut microbiota underlies variability in susceptibility to weight gain in human patients. Furthermore, there is no reason to believe that olanzapine is unique in this respect: weight gain is a common adverse effect of numerous drugs [58], many of which undergo enterohepatic circulation. The gut microbiota represents both a biomarker and potential therapeutic target for drug-induced weight gain.

## Supporting Information

S1 Figure **Survey of effects of orally-administered olanzapine (OLZ) on eight inbred mouse strains.** (**A**) Weight gain during 80 days of OLZ treatment versus placebo in female mice from eight inbred laboratory strains (*n* = 4 OLZ-treated and 4 placebo-treated mice per panel). OLZ was compounded into mouse feed at 50 mg/kg, and all mice were fed a high-fat diet *ad libitum*. (**B**) Plasma OLZ levels after 18 weeks of treatment, by strain.
(EPS)

S2 Figure **Dose titration for oral administration of olanzapine in female C57BL/6J mice.** (**A**) Plasma olanzapine (OLZ) concentration measured by LC-MS/MS (*49*) *vs.* concentration in mouse food (*n* = 5 subjects). Grey shaded region indicates target dose in human patients. (**B**) Dose-response relationship for OLZ and two metabolic parameters, body weight (in grams) and adiposity (fat mass as percentage of total body weight). Points indicate rate of increase per day in each metabolic parameter, estimated by ordinary least-squares regression. Vertical bars represent 95% confidence intervals (obtained by likelihood profiling) for the drug-effect estimates.
(EPS)

S3 Figure **Effects of olanzapine (OLZ) treatment on weight gain and adiposity.** (**A**) Weight gain over 9 weeks in female C57BL/6J mice fed a high-fat diet (HFD) *ad libitum*. Each line represents a single mouse (*n* = 24 subjects); mice were 6 weeks of age at initiation of HFD. Grey shaded intervals indicate period of OLZ treatment. (**B**) Adiposity (percent body fat) during HFD-OLZ versus HFD-placebo phase at matched time points (*n* = 48 or 2 observations ×24 subjects). (**C**) Adiposity (percent body fat) versus body weight during HFD-placebo (open circles) or HFD-OLZ (filled circles) phase. Pearson's r = 0.819 (95% CI 0.697–0.895) on *n* = 72 (3 observations ×24 subjects).
(EPS)

S4 Figure **Inter-individual variability in effect of olanzapine (OLZ) on weight gain (** ***n*** **= 24 subjects).** At left, confidence intervals for posterior mode of subject-specific offset (eg. baseline weight differences); at right, confidence intervals for subject-specific OLZ effect, which represent deviations from the overall mean OLZ effect.
(EPS)

S5 Figure **Between-subjects (** ***β*** **) diversity by unweighted UniFrac (** ***n*** **= 95 samples  = 4 time points ×24 subjects – 1 failed sample), visualized using non-metric multidimensional scaling.** Panels are split by study arm (see **Fig. 1C**) and observations on the same subject are connected by a grey line.
(EPS)

S6 Figure **Time series of gut microbiota composition at the individual level.** Colored regions show relative abundance, per subject, of specific bacterial classes at each of the experiment's four time points (see **Fig. 1C**). Subjects are grouped by study arm (left, A; right, B) and cage (numbered sequentially, 1–6) as indicated in the bar above each panel.
(EPS)

S7 Figure **Rarefaction curves for assessing coverage of 16S rRNA sequencing.** Each curve plots the number of OTUs discovered (species richness; y-axis) as a function of sequencing depth (reads per sample; x-axis) in a single sample (*n* = 95 samples  = 4 time points ×24 subjects – 1 failed sample). Curves are grouped by feces-collection time point (see **Fig. 1C**) and treatment status.
(EPS)

S8 Figure **Maximum-likelihood phylogeny of operational taxonomic units (OTUs) identified by 16S rDNA sequencing, unrooted (** ***n*** **= 8067 OTUs).** Scale for branch lengths is indicated by scale bar at lower-right, in units of nucleotide substitutions per site.
(EPS)

S9 Figure **Heatmap of (Pearson's r) correlation matrix for 500 most-variable OTUs, colored from blue (r = +1) through white (r = 0) to red (r = −1).** Rows and columns are hierarchically clustered in order to reveal block structure.
(PNG)

S1 Table **Sample metadata for 96 fecal samples.** Timepoints are coded in order according to **Fig. 1C**.
(XLSX)

S2 Table **Raw operational taxonomic unit (OTU) abundance table.** Sample identifiers follow those in **S1 Table**.
(XLSX)

S3 Table **Taxonomic identification of operational taxonomic units (OTUs) to the rank of family.** Missing data are coded as “NA.” OTU identifiers follow those in **S2 Table**.
(XLSX)

S4 Table **Coefficient estimates for effect of olanzapine and high-fat diet on abundance of specific operational taxonomic units (OTUs).** OTU identifiers follow those in **S2 Table**. Columns are as follows: Beta, the estimated effect on the log scale (see S1 Notes); 95% CI, confidence bounds for the effect at nominal significance level *α* = 0.05; Term, the effect being tested (drug or diet); *p*-value, nominal *p*-value from likelihood-ratio test of null hypothesis that the true effect is zero; FDR-adjusted *p*-value, nominal *p*-value adjusted for multiple-testing by the method of Benjamini and Hochberg.
(XLSX)

S1 Notes **Details of bioinformatics analyses and statistical methods.**
(DOCX)
