# Supplementary figures and images for: The Antipsychotic Olanzapine Interacts with the Gut Microbiome to Cause Weight Gain in Mouse
Source: PLoS One. 2014 Dec 15;9(12):e115225. doi: 10.1371/journal.pone.0115225 (PMC4266663; doi:10.1371/journal.pone.0115225)

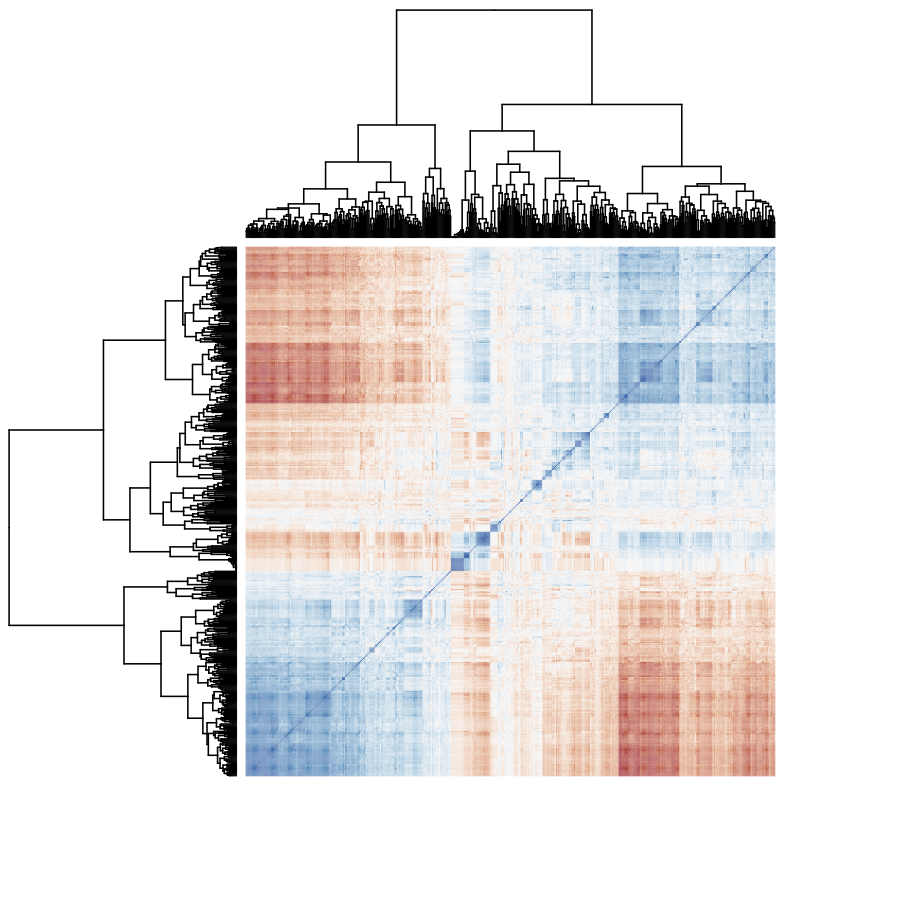

Supplement: S9 Figure — Heatmap of (Pearson's r) correlation matrix for 500 most-variable OTUs, colored from blue (r = +1) through white (r = 0) to red (r = −1). Rows and columns are hierarchically clustered in order to reveal block structure. (PNG) [file pone.0115225.s009.png]
