# Supplementary material for: The Antipsychotic Olanzapine Interacts with the Gut Microbiome to Cause Weight Gain in Mouse
Source: PLoS One. 2014 Dec 15;9(12):e115225. doi: 10.1371/journal.pone.0115225 (PMC4266663; doi:10.1371/journal.pone.0115225)
Supplement: S1 Notes — Details of bioinformatics analyses and statistical methods. (DOCX) [file pone.0115225.s014.docx]

# Supplementary materials:

Figures S1-S9

Tables S1-S4

Supplementary notes

**
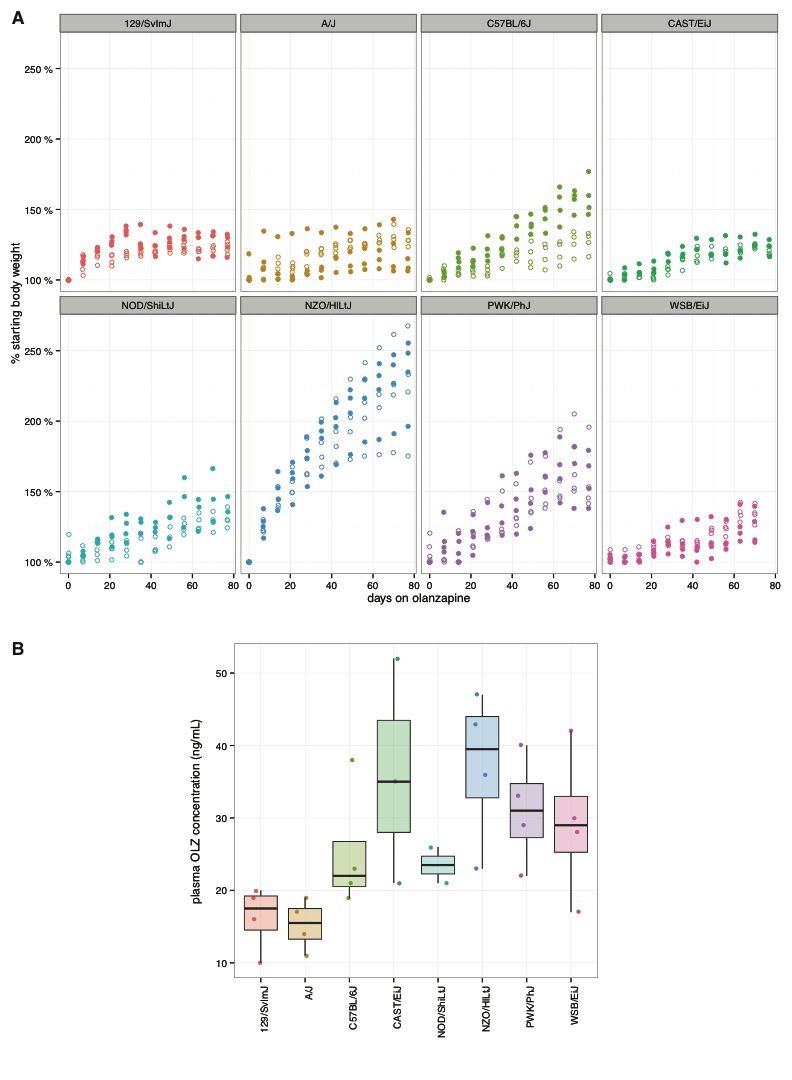
**

**Fig. S1:** Survey of effects of orally-administered olanzapine (OLZ) on eight inbred mouse strains. **(A)** Weight gain during 80 days of OLZ treatment versus placebo in female mice from eight inbred laboratory strains (*n* = 4 OLZ-treated and 4 placebo-treated mice per panel). OLZ was compounded into mouse feed at 50 mg/kg, and all mice were fed a high-fat diet *ad libitum*. **(B)** Plasma OLZ levels after 18 weeks of treatment, by strain.

**
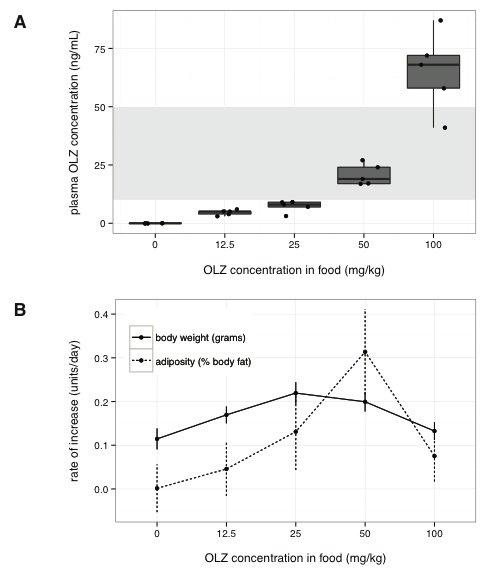
**

**Fig. S2:** Dose titration for oral administration of olanzapine in female C57BL/6J mice. **(A)** Plasma olanzapine (OLZ) concentration measured by LC-MS/MS (*49*) *vs.* concentration in mouse food (*n* = 5 subjects). Grey shaded region indicates target dose in human patients. **(B)** Dose-response relationship for OLZ and two metabolic parameters, body weight (in grams) and adiposity (fat mass as percentage of total body weight). Points indicate rate of increase per day in each metabolic parameter, estimated by ordinary least-squares regression. Vertical bars represent 95% confidence intervals (obtained by likelihood profiling) for the drug-effect estimates.

**
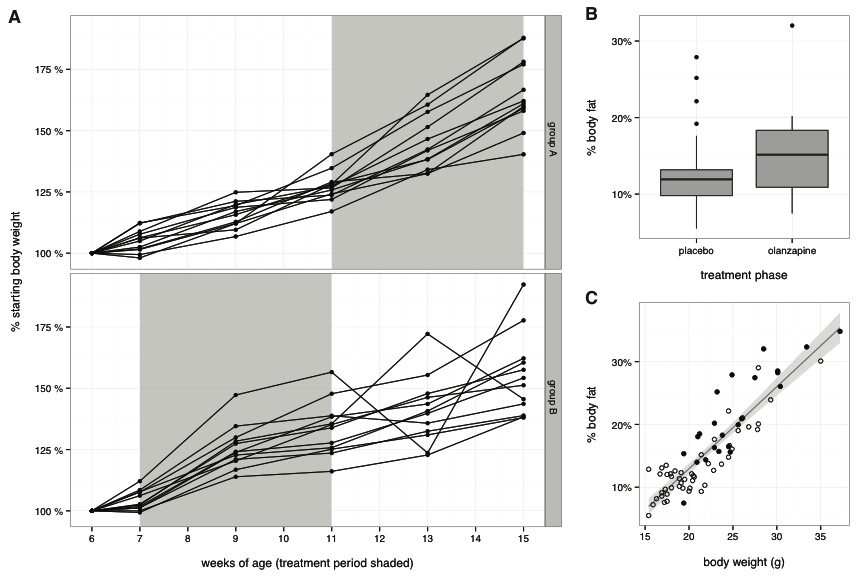
**

**Fig. S3:** Effects of olanzapine (OLZ) treatment on weight gain and adiposity. **(A)** Weight gain over 9 weeks in female C57BL/6J mice fed a high-fat diet (HFD) *ad libitum*. Each line represents a single mouse (*n* = 24 subjects); mice were 6 weeks of age at initiation of HFD. Grey shaded intervals indicate period of OLZ treatment. **(B)** Adiposity (percent body fat) during HFD-OLZ versus HFD-placebo phase at matched time points (*n* = 48 or 2 observations × 24 subjects). **(C)** Adiposity (percent body fat) versus body weight during HFD-placebo (open circles) or HFD-OLZ (filled circles) phase. Pearson’s r = 0.819 (95% CI 0.697-0.895) on *n* = 72 (3 observations × 24 subjects).

**Fig. S4:** Inter-individual variability in effect of olanzapine (OLZ) on weight gain (*n* = 24 subjects). At left, confidence intervals for posterior mode of subject-specific offset (eg. baseline weight differences); at right, confidence intervals for subject-specific OLZ effect, which represent deviations from the overall mean OLZ effect.

**Fig. S5:** Between-subjects (*β*) diversity by unweighted UniFrac (*n* = 95 samples = 4 time points × 24 subjects – 1 failed sample), visualized using non-metric multidimensional scaling. Panels are split by study arm (see **Figure 1C**) and observations on the same subject are connected by a grey line.

**Fig. S6:** Time series of gut microbiota composition at the individual level. Colored regions show relative abundance, per subject, of specific bacterial classes at each of the experiment’s four time points (see **Figure 1C**). Subjects are grouped by study arm (left, A; right, B) and cage (numbered sequentially, 1-6) as indicated in the bar above each panel.

**Fig. S7:** Rarefaction curves for assessing coverage of 16S rRNA sequencing. Each curve plots the number of OTUs discovered (species richness; y-axis) as a function of sequencing depth (reads per sample; x-axis) in a single sample (*n* = 95 samples = 4 time points × 24 subjects – 1 failed sample). Curves are grouped by feces-collection time point (see **Figure 1C**) and treatment status.

**Fig. S8:** Maximum-likelihood phylogeny of operational taxonomic units (OTUs) identified by 16S rDNA sequencing, unrooted (*n* = 8067 OTUs). Scale for branch lengths is indicated by scale bar at lower-right, in units of nucleotide substitutions per site.


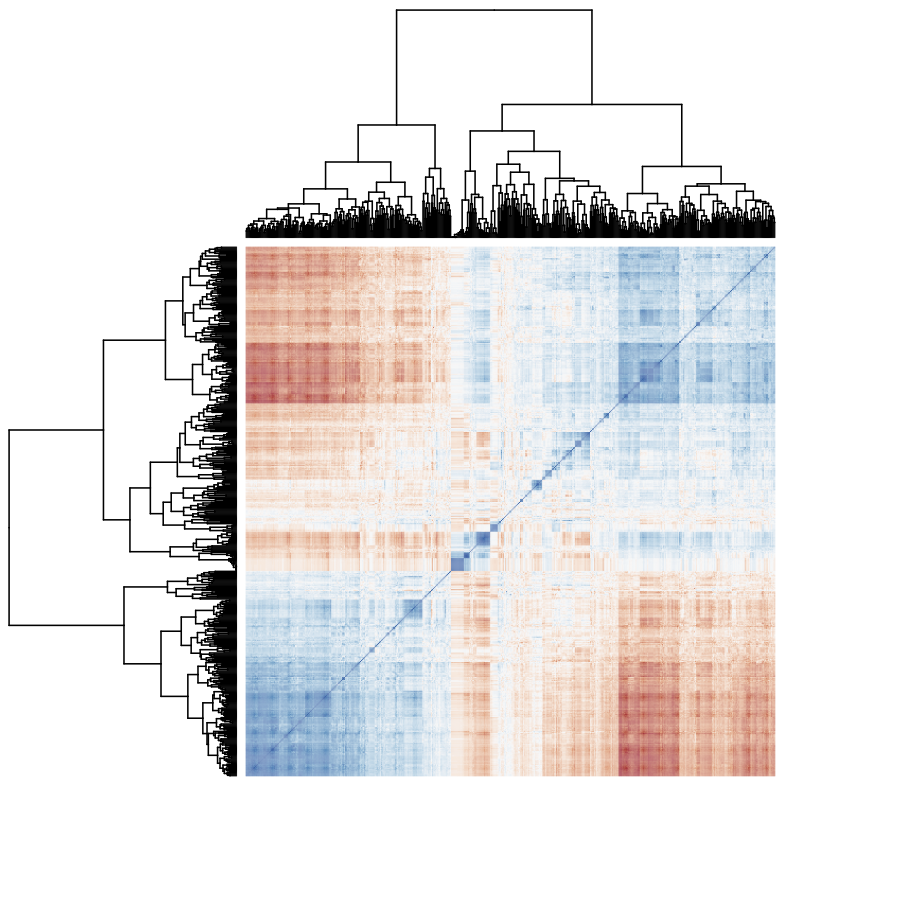


**Fig. S9:** Heatmap of (Pearson’s r) correlation matrix for 500 most-variable OTUs, colored from blue (r = +1) through white (r = 0) to red (r = -1). Rows and columns are hierarchically clustered in order to reveal block structure.

**Table S1:** Sample metadata for 96 fecal samples. Timepoints are coded in order according to **Figure 1C**.

**Table S2:** Raw operational taxonomic unit (OTU) abundance table. Sample identifiers follow those in **Table S1**.

**Table S3:** Taxonomic identification of operational taxonomic units (OTUs) to the rank of family. Missing data are coded as “NA.” OTU identifiers follow those in **Table S2**.

**Table S4:** Coefficient estimates for effect of olanzapine and high-fat diet on abundance of specific operational taxonomic units (OTUs). OTU identifiers follow those in **Table S2**. Columns are as follows: Beta, the estimated effect on the log scale (see supplementary notes); 95% CI, confidence bounds for the effect at nominal significance level *α* = 0.05; Term, the effect being tested (drug or diet); *p*-value, nominal *p*-value from likelihood-ratio test of null hypothesis that the true effect is zero; FDR-adjusted *p*-value, nominal *p*-value adjusted for multiple-testing by the method of Benjamini and Hochberg.

***Note:*** *because of their large size, supplementary tables are provided as separate Excel files.*

# Supplementary notes

**Quality-control for gut microbiome survey.** Gut bacteria were surveyed by amplification and sequencing of the fourth hypervariable region (V4) of the 16S ribosomal RNA subunit gene from each fecal sample. Sequence reads were binned into operational taxonomic units (OTUs) which were identified to the taxonomic rank of family. Adequacy of sequencing depth was assessed using rarefaction curves (**Figure S7**); a median 15,461 unique template sequences (*52*) were identified per sample (range 6997 – 32 552). A total of 8067 OTUs were identified across all samples (displayed on the phylogenetic tree in **Figure S8**.) Of these, a median 612 (range 407 – 1010) were present in any given sample. Sample metadata are provided in **Table S1**, the unrarefied OTU abundance matrix in **Table S2** and taxonomic assignments for each OTU in **Table S3**.

**Assessment of between-samples (*β*) microbial diversity.** Both the weighted and the unweighted UniFrac metrics were used to examine patterns of between-sample diversity in gut microbiome composition. Weighted UniFrac results, which are sensitive to both presence/absence and relative abundance of OTUs, are presented in the main text. The unweighted UniFrac distance matrix, which takes into account only the presence or absence of each OTU in each sample, was also subjected to non-metric multidimensional scaling (*58*) and two axes explained 98% of variance in microbial community composition (**Figure S5**). Effects of experimental covariates were assessed by permutational non-parametric multivariate ANOVA (PERMANOVA) (*57*) and the results are shown in **Table 1**. The effect of the high-fat diet was most pronounced, accounting for 16.5% of variance (permutation *p* < 1.00 × 10^-4^). The effect of olanzapine treatment was also significant and accounted for 1.4% of variance (permutation *p* = 0.021). Both effects were highly variable between mice (**Figure S5**). Cage and temporal effects, nuisance covariates in our experiment, accounted for 14.3% of variance. Within-groups dispersion was greater among observations on high-fat diet *vs.* regular chow (*p* = 1.0 × 10^-5^).

**Modelling abundance of specific OTUs.** Like many other techniques that rely on high-throughput tag sequencing, 16S rDNA-based microbiome surveys are subject to biases arising due to very sparse sampling and differential sequencing depth across samples. In order to mitigate these biases raw OTU counts were normalized prior to testing for significant differences in absolute abundance between samples. For this purpose we used the dynamic-quantile normalization method implemented in the R package metagenomeSeq (*59*). We identified a total of 8067 OTUs across 96 fecal samples, but only a median of 612 were present in any single sample. In order to avoid biases arising due to separation, we restricted our analysis to 1127 OTUs present (at normalized abundance > 0) in at least 12 samples.

Diet and drug effects at the level of individual OTUs were then assessed using Poisson generalized linear mixed-effects models (GLMMs) in order to accommodate both within-subjects correlation due to the repeated-measures aspect of the experimental design and between-subjects correlation arising from co-housing. Subject and cage were treated as nested random effects, and timepoint as an additional random effect. Diet and drug were treated as fixed effects. The hypotheses of no diet effect and no drug effect were tested via standard likelihood-ratio tests with degrees of freedom adjusted by the Kenward-Roger approximation (*60*) as implemented in the R package pbkrtest (<http://cran.r-project.org/web/packages/pbkrtest/>). In the Poisson GLMM framework, coefficients are on the log scale.

Of 1127 testable OTUs, 606 (53.8%) were differentially abundant between regular chow and high-fat diet phases and 512 (45.4%) were differentially abundant between high-fat diet-placebo and high-fat diet-olanzapine treatment phases at false discovery rate (FDR) ≤ 0.05 (**Table S4**). (Note that an FDR-adjusted *p*-value is the largest FDR at which the associated nominal *p*-value would be declared significant.) Class Clostridia was enriched among OTUs with a diet effect ($\chi_{1}^{2}$ = 74.8, FDR-adjusted *p* = 5.90 × 10^-19^), but no class was enriched to a statistically significant level among OTUs with a drug effect. Pairwise correlations between abundances of the 500 most-variable OTUs over the course of the experiment are visualized as a heatmap in **Figure S9** to reveal potential functional “communities” within the microbiota.
